# Biomimetic Redox-Responsive Mesoporous Organosilica Nanoparticles Enhance Cisplatin-Based Chemotherapy

**DOI:** 10.3389/fbioe.2022.860949

**Published:** 2022-03-16

**Authors:** Fangman Chen, Fan Zhang, Yanbin Wang, Jiahui Peng, Lei Cao, Qian Mei, Mingfeng Ge, Li Li, Meiwan Chen, Wen-fei Dong, Zhimin Chang

**Affiliations:** ^1^ School of Biomedical Engineering (Suzhou), Division of Life Sciences and Medicine, University of Science and Technology of China, Hefei, China; ^2^ CAS Key Laboratory of Bio Medical Diagnostics, Suzhou Institute of Biomedical Engineering and Technology Chinese Academy of Sciences, Suzhou, China; ^3^ Nephrology Department of the Fourth Affiliated Hospital of XinJiang Medical University, Macau, China; ^4^ State Key Laboratory of Quality Research in Chinese Medicine, Institute of Chinese Medical Sciences, University of Macau, Macau, China

**Keywords:** cisplatin, mesoporous silica nanoparticles, glutathione depletion, biomimetic nanocarrier, degradation

## Abstract

Cisplatin-based chemotherapy is dominated in several cancers; however, insufficient therapeutic outcomes and systemic toxicity hamper their clinical applications. Controlled release of cisplatin and reducing inactivation remains an urgent challenge to overcome. Herein, diselenide-bridged mesoporous organosilica nanoparticles (MON) coated with biomimetic cancer cell membrane were tailored for coordination responsive controlled cisplatin delivery and GSH depletion to strengthen Pt-based chemotherapy. Cisplatin-loaded MON (MON-Pt) showed high loading capacity due to robust coordination between selenium and platinum atoms and preventing premature leakage in normal tissue. MON-Pt exhibited a controlled release of activated cisplatin in response to the redox tumor microenvironment. Meanwhile, MON-Pt containing redox-responsive diselenide bonds could efficiently scavenge intracellular inactivation agents, such as GSH, to enhance Pt-based chemotherapy. 4T1 breast cancer cell membranes cloaked MON-Pt (MON-Pt@CM) performed efficient anticancer performance and low *in vivo* system toxicity due to long blood circulation time and high tumor accumulation benefiting from the tumor targeting and immune-invasion properties of the homologic cancer cell membrane. These results suggest a biomimetic nanocarrier to control release and reduce the inactivation of cisplatin for efficient and safe Pt-based chemotherapy by responding and regulating the tumor microenvironment.

## Introduction

Cancer has become the leading cause of mortality worldwide (Ferlay et al., 2021). Chemotherapy is an essential tool for fighting against cancer (Darge et al., 2021; Wang et al., 2021). Since cisplatin was approved in 1978 by the FDA, platinum-based chemotherapy, including oxaliplatin and carboplatin, is approved commonly for treating numerous cancers that occur in breast, cervix, colon, ovaries, and lung (He et al., 2019; Lin et al., 2020; Chen et al., 2021). The outcome of clinical cisplatin-based chemotherapy becomes inefficient in patients because of serum albumin in blood deactivating cisplatin, inefficient cellular uptake by cancer cells, and easy inactivation by intracellular metallothionein (MT) and glutathione (GSH) (Han et al., 2018; Lan et al., 2018; Kawahara et al., 2019; Zeng et al., 2020). Moreover, cisplatin kills cancer cells and affects normal cells due to lack of selectivity (Davidi et al., 2018; Huang et al., 2018). Consequently, cisplatin inevitably causes severe side effects such as nephrotoxicity and neurotoxicity as well as toxicity to the gastrointestinal tract (Davidi et al., 2018; Huang et al., 2018; Fujishiro et al., 2021; Sun et al., 2021). Cisplatin-based chemotherapy is dominated in several cancers; however, poor therapeutic outcomes and systemic toxicity hamper their clinical applications (Fujishiro et al., 2021; Kuo et al., 2021). It is imperative to design an elaborate drug delivery system to overcome unwanted side effects and deactivation of cisplatin-based chemotherapy.

Drug delivery carriers could endow a controlled and on-demand drug release manner in response to a different stimulus such as pH, redox, light, and magnetic fields (Shao et al., 2018b; Cai et al., 2019; Wang H. et al., 2020; Wang Y. et al., 2020; Chen et al., 2020; Shao et al., 2020). Acid-sensitive nanocarriers, such as polymer micelles and inorganic particles, are widely employed to deliver Pt-based therapeutic agents. However, the encapsulation of cisplatin through charge interaction results in low loading capacity (Wright et al., 2019; Khan et al., 2020). To improve the loading capacity, the coordination coupling of the carboxyl group and cisplatin are developed to achieve high loading capacity (10–20 wt%) and tumor microenvironment-responsive release of cisplatin. Such a coordination binding avoids premature cisplatin leakage in complicated physiological environmental conditions (He et al., 2014; Liu et al., 2021a). Although these nanocarriers show high drug loading capacity and an on-demand drug release manner, it is difficult to prevent the inactivation of cisplatin in high redox tumor microenvironments. There is increasing evidence that cisplatin is quickly deactivated by intracellular metallothionein (MT) and glutathione (GSH) (Han et al., 2018; Lan et al., 2018; Zeng et al., 2020). The intracellular GSH (10 mM) in cancer tissue is 100- to 1000-fold higher than normal tissue (Yin et al., 2018), limiting the performance of cisplatin-based chemotherapy (Ling et al., 2018; Xu et al., 2019). Thus, it poses a challenge for cisplatin delivery to integrate high loading capacity, controlled drug release, and reversion of deactivation into one of the simplified nanocarriers.

Selenium (Se) as an essential element participates in number of critical biological processes, including maintaining the redox homeostasis in humans (Liu et al., 2021b). Selenium-containing materials have attracted much attention for multiresponsive drug delivery and anticancer activities (Sun et al., 2020; Birhan and Tsai, 2021). We recently developed a diselenide-bridged MONs that showed great potential as redox-responsive drug-delivery vehicles for controlled drug release in special tumor environments (Shao et al., 2018a; Chengxin Shi, 2022; Peng et al., 2022; Yang et al., 2022). Diselenide-bridged MONs achieved biodegradation in a high concentration of reductive GSH solution while scavenging; meanwhile, scavenged intracellular GSH. Besides its unique redox properties, selenium exhibits robust coordination with transition metals elements (Lee et al., 2018; Dias et al., 2020). Currently, selenium-containing polymers are used to deliver metal-based chemotherapeutics (e.g., platinum and ruthenium compounds) utilizing their coordination properties. With these findings in mind, we propose that diselenide-bridged MONs could be an ideal carrier for cisplatin delivery to achieve high loading capacity, controlled drug release, and sufficient reversion of deactivation.

**SCHEME 1:**
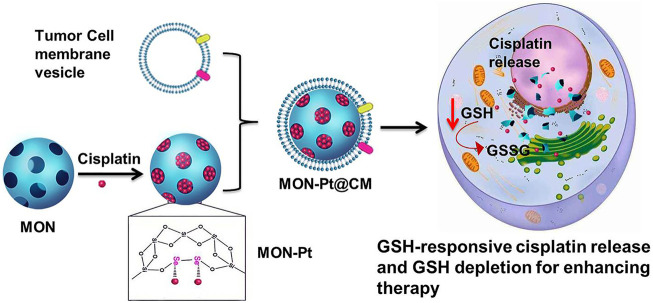
Schematic of synthesis of diselenide bond-bridged MON for redox-responsive cisplatin delivery and GSH depletion for efficient and safe Pt-based chemotherapy.

Herein, we prepared diselenide-bridged MONs to deliver cisplatin for highly efficient and safe chemotherapy. The diselenide-bridged MONs achieved a high loading capacity of cisplatin (MON-Pt) by coordination binding between active cisplatin and selenium-atom in MONs (Scheme 1). MON-Pt exhibited a controlled manner of cisplatin release in response to the redox tumor microenvironment, and coordination binding avoided the premature leakage of cisplatin in normal tissue. Meanwhile, MON-Pt could efficiently achieve GSH depletion in the responsive release of cisplatin. To improve targeting tumor and immune-invasion, biomimetic nanocarrier (MON-Pt@CM) was constructed via coating MON-Pt with the cancer cell membrane (CM), exhibiting long blood circulation time and high tumor accumulation. The *in vitro* and *in vivo* results have validated the advantages of this biomimetic MON-Pt@CM that reduced server-side effects and improved therapeutic potency.

## Materials and Methods

### Chemicals and Reagents

Chemicals and characterization method was details in Supporting information.

### Preparation of MON

According to our previous reports, a designed diselenide-bridged MON was synthesized (Shao et al., 2018a). Briefly, 0.6 g cetyltrimethylammonium tosylate (CTAT), and 0.15 g triethanolamine (TEA) were added into 40 ml deionized water. After stirring at 80°C for 30 min, a mixture of 4.0 g TEOS, 1.0 g Bis [3-(triethoxysilyl)propyl]diselenide (BTESePD) and 3 ml ethanol was added dropwise. The mixture was a continuous reaction for another 4 h. The PEG-silane (3 g) was added dropwise into the mixture after cooling to room temperature, and the mixture was stirred overnight. Then, the mixture was stirred for another 4 h at 80°C. The products were collected, washed thrice, and extracted with a solution of NH_4_NO_3_ (0.7% w/v) in ethanol for 12 h. FITC-labeled MON was obtained for cellular internalization tracking as our previous works (Shao et al., 2018b). The nonbiodegradable MSN was prepared according to the above mothed except TEOS (5 g) as the only silica source.

### Preparation of Activated Cisplatin

The activated cisplatin was prepared as described in the literature (Sood et al., 2006). Cisplatin (400 mg, 1.33 mmol) and AgNO_3_ (406 mg, 2.39 mmol) were added into 10 ml H_2_O. Subsequently, a diluted HNO_3_ solution was used to tune pH value to 2. The suspension was stirred at 70°C overnight in the dark. The mixture was cooled at 4°C overnight. The mixture was filtrated through a 0.22 μm syringe filter to obtain activated cisplatin.

### Cisplatin Loading and GSH-Responsive Drug Release

A 20 mg MON (or MSN) was mixed with 8 mg active cisplatin in 10 ml solution under sonication, and the resulting mixture was stirred at 500 rpm at 37°C overnight to obtain MON-Pt. The amount of platinum was analyzed by inductively coupled plasma atomic emission spectrometry (ICP-OES). The drug-loading content was calculated as in the equation, Drug loading content (%) = mass of cisplatin in MON-Pt/mass of MON-Pt. For drug release analysis, MON-Pt (5 mg) was dispersed in 10 ml PBS with/without 10 mM GSH has shaken at 100 rpm. The amount of cisplatin release in the supernatant was measured.

### Cancer Cell Membrane Derivation and MON-Pt@CM Preparation

The cancer cell membrane–derived vesicles were prepared as described previously (Zhang et al., 2021). 4T1 cells were collected, lysed, and collected via centrifugation. Cell membrane fragments were obtained by extrusion and sonication. The final CM vesicles were extruded serially through 1-μm, 400-nm, and 200-nm polycarbonate membranes (Whatman) with an Avanti Mini-Extruder (Avanti Polar Lipids). The 4-chlorobenzenesulfonate salt (DiD) dye-labeled CM vesicles were prepared by staining 4T1 cancer cells before hypotonic lysing. The obtained CM mixed with MON-Pt, sonicated for 5 min to obtain cancer cell membrane-coated MON-Pt (MON-Pt@CM). The obtained MON-Pt@CM was stored at 4°C. SDS-PAGE was used to analyze the protein component of MON-Pt@CM. The marker protein CD47 of 4T1 cells in 4T1 cell membranes and MON-Pt@CM was identified by Western blot.

### Extracellular GSH Depletion Assay

The MON was mixed with GSH (10 mM) in a 10 ml solution. The mixtures were shaken overnight at room temperature, and the GSH concentration of the supernatant was analyzed by Ellman’s reagent.

### Cell Viability

The cell viability was determined by 3-(4,5-dimethylthiazole-2-yl)-2,5-diphenyl tetrazolium bromide (MTT) assay. Briefly, 4T1 cells (5×10^3^ cells/well) were inoculated overnight in a 96-well plate. The next day, cells were treated with different formulations. After treatment for 48 h, cell cytotoxicity was analyzed.

### Intracellular GSH Depletion Assay

4T1 cells (3×10^4^ cells/well) were cultured in 24-well culture plate overnight. After treating with formulations for 24 h, cells were washed, and intracellular glutathione content was analyzed by the Micro Reduced Glutathione (GSH) Assay Kit (BC1175, Solarbio, Beijing, China), following the manufacturer’s protocol.

### Cellular Uptake and Homogenous Targeting Effects

Homogenous targeting capacity of cisplatin, MON-Pt or MON-Pt@CM (30 μg/ml based on cisplatin) was measured. 4T1, MCF-10A, and RAW264.7 cells (2×10^5^ cells/well) were inoculated in a six-well plate for quantifying analysis of endocytosis. After being inoculated overnight, cells were treated with formulations for another 5 h. The cells were digested and collected. The cellular uptake of MON-Pt@CM was measured by analysis of platinum content using ICP-OES.

4T1 cells (3×10^4^ cells/well) were inoculated overnight in a 24-well culture plate to analyze the colocalization of the cancer cell membranes and MON cores. After incubation with FITC-MON@CM-DiD (30 μg/ml) for 3 h, 4T1 cells were washed and stained with DAPI. The cell imaging was observed under a fluorescence microscope.

### Pharmacokinetics and Biodistribution *in vivo*

The ethical committee approved all experimental animal protocols, according to the Suzhou Institute of Biomedical Engineering and Technology recommendations, the Chinese Academy of Sciences Laboratory Animal Center. 4T1 tumor model was established by subcutaneous injection of 4T1 cells (5 × 10^5^ cells) into the left flank of the BALB/C mice (18–20 g, 4–5 weeks). Animals were cultivated in specific pathogen-free (SPF) conditions. To investigate the pharmacokinetics and biodistribution, 4T1 tumor-bearing mice were injected with free cisplatin, MON-Pt, or MON-Pt@CM solution (2 mg/kg based on cisplatin). ICP-OES measured the platinum content in the blood. Major organs and tumor were harvested to investigate the organ distribution after administration of 24 h. ICP-OES measured the platinum content in each sample.

### *In vivo* Chemotherapy

The 4T1 tumor model was established. When the tumor volume was grown to 100 mm^3^, the mice were administered with PBS, cisplatin, MON-Pt, MON-Pt@CM, MSN-Pt@CM (2 mg/kg based on cisplatin) once every 3 days. All of the mice had recorded tumor volume and body weight. After treatment for 21 days, the mice were sacrificed and recorded tumor weight. The main organs (liver, spleen, kidneys, heart, and lungs) were stained with Hematoxylin and eosin (H&E) to analyze pathophysiology. Biochemical parameters indexes were also analyzed.

## Results and Discussion

### Preparation and GSH Depletion of MON

**FIGURE 1:**
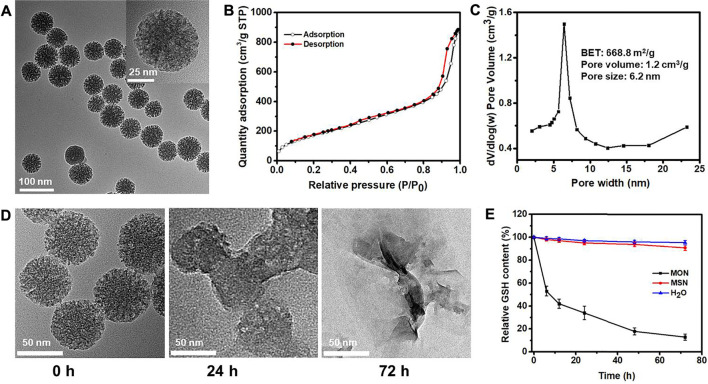
Preparation and characterization of MON. **(A)** TEM, **(B)** N_2_ adsorption isotherm,**(C)** pore size distribution of MON. **(D)** TEM images of MONs showing degradation at 0, 24 and 72 h under 10 mM GSH. **(E)** GSH depletion of MON.

Mesoporous organosilica nanoparticles with high biocompatibility, porous structure, tunable morphology, highly efficient drug loading capacity, and facile surface-functionalization are widely used to deliver anticancer drugs and perform controlled drug release in response to the tumor microenvironment (Chen et al., 2018; Omar et al., 2018; Yu et al., 2018; Zhang et al., 2020). According to previous reports, diselenide-bridged MON were fabricated via the sol-gel method (Shao et al., 2020; Zhang et al., 2021). The morphology and pore structures of MON were characterized by transmission electron microscope (TEM) and scanning electron microscope (SEM). It was shown that parent MON exhibited uniform spherical particles with an average diameter of 60 ± 5 nm (Figure 1A, and Supplementary Figure S1). The MON showed worm-like mesoporous channels. The characteristic type-IV of N_2_ adsorption–desorption isotherms indicate a mesoporous structure (Figure 1B), and the average pore size was 6.2 nm according to the BJH pore size distribution (Figure 1C). The MON showed a high surface area (668.8 m^2^/g) and cumulative pore volume (1.2 cm^3^/g), beneficial for drug loading.

Energy-dispersive X-ray spectroscopy (EDS) revealed a strong signature for the Se element in the MON (Supplementary Figure S2). This agrees with the high Se element content (12.4%) in diselenide-bond-bridged MON determined by ICP-OES. The high hybrid of diselenide bond in MON confers sensitive matrix-degradation in response to redox conditions. The degradation of MON was investigated in the media mimicking the tumor microenvironment (10 mM GSH). We observed that MON underwent rapid degradation after 1 day of incubation (Figure 1D). The structure of MON disintegrated into small fragments after 3 days of exposure to GSH solution. However, MON was stable in normal physiological conditions (Supplementary Figure S3). Our previous research reveals the degradation mechanism attributed to the diselenide bond cleavage under reductive conditions. We further confirmed the mechanisms by determining the residual content of GSH in the mimicking media. As shown in Figure 1E, the content of reductive GSH was rapidly decreased to 13.5% after 72 h, indicating excellent GSH depletion. As a control, nondegradable mesoporous silica nanoparticles (MSN) were prepared and exhibited a stable structure in GSH solution (Supplementary Figure S4) and did not cause consumption of GSH (Figure 1E). These results verify that diselenide-bond-bridged MON has a special GSH scavenge, which may avoid the deactivation of cisplatin-based chemotherapy in the tumor microenvironment.

### Preparation and Control Release of MON-Pt

It is well-established that cisplatin exists as an equilibrium of “neutral” or “cationic” structure in an aqueous solution (Liu et al., 2021a). The “neutral” structure is dominant in extracellular high Cl^−^ ion concentration (≈150 mM). The cationic structure is pharmaceutically active cisplatin due to Cl^−^ ion concentration (≈30 mM), which induced nuclear DNA crosslinking. This activated cisplatin was easily deactivated by a high intracellular concentration of GSH in tumor cells. Meanwhile, there are also some advantages of cationic cisplatin with coordination capacity for high drug loading and controllable delivery. These findings inspired us to consider loading cationic, activated cisplatin into diselenide-bond-bridged MON for controllable delivery and scavenging intracellular GSH to enhance Pt-based chemotherapy.

**FIGURE 2:**
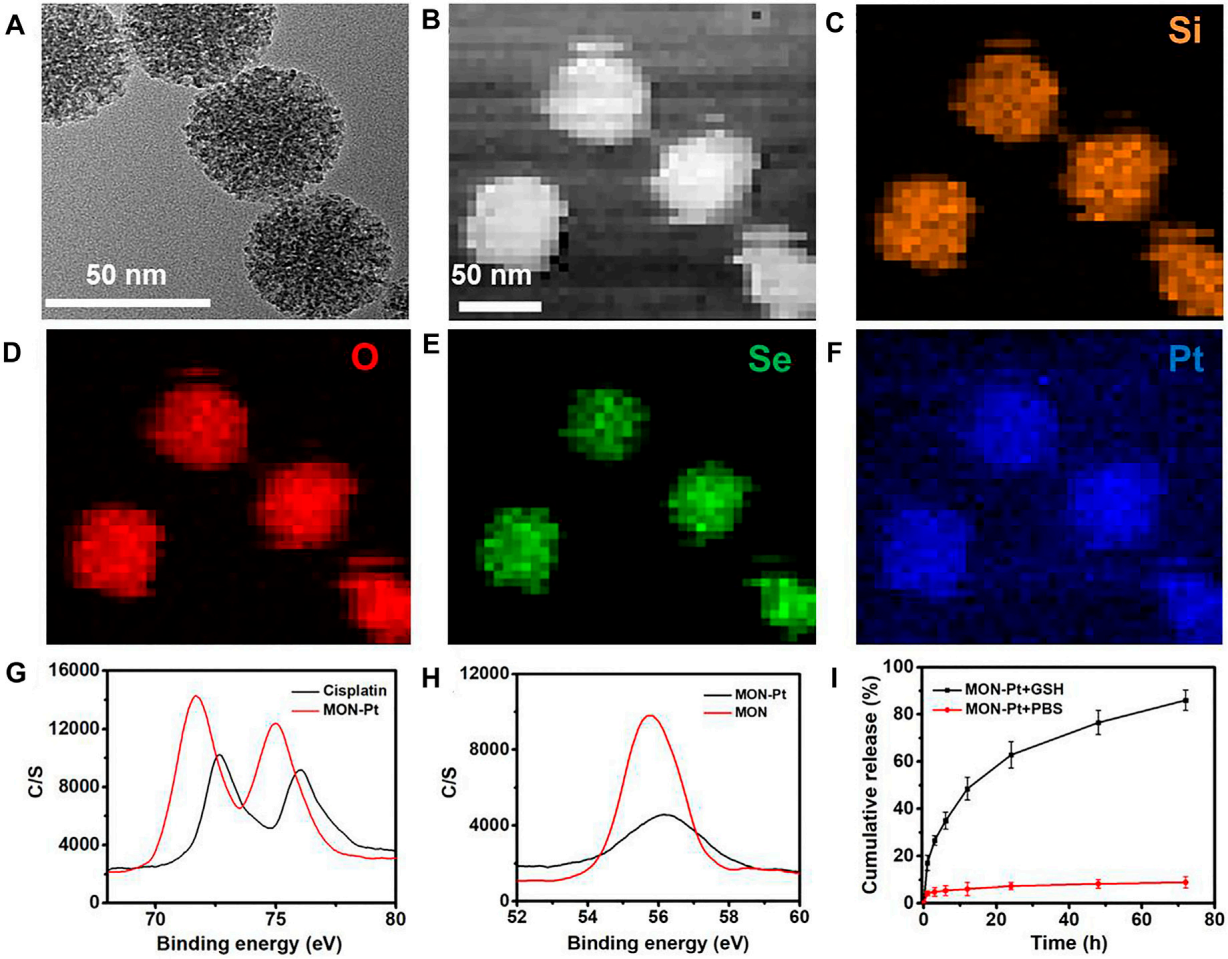
Preparation and characterization of MON-Pt. **(A)** TEM of MON-Pt. **(B–F)** Mapping images of MON-Pt. XPS spectrum of **(G)** platinum and **(H)** selenium of MON-Pt. **(I)** Cisplatin release profiles of MON@Pt in the presence or absence of 10 mM GSH. All data are mean ± SD (n = 3).

The active cisplatin was synthesized by silver nitrate and collected through centrifugation and filtration. The high hybrid of selenium atom in MON provided an abundant coordination site. MON loading cisplatin (MON-Pt) was prepared via mixing with active cisplatin, resulting in 16.1% ± 0.7% of the loading capacity of cisplatin. The morphology of MON-Pt was consistent with parent MON (Figure 2A). The elemental mapping of MON-Pt revealed a strong signal of Pt and Se elements (Figures 2B–F). In addition, the binding energy of selenium and platinum atoms was characterized by X-ray photoelectron spectroscopy (XPS). After loading with cisplatin, the binding energy of selenium 3 days in MON-Pt shifted upward from 55.7 to 56.2 eV (Figure 2G). Meanwhile, the binding energy of platinum 3p in MON-Pt shifted downward from 75.9 to 72.3 to 74.9 and 71.7 eV, respectively (Figure 2H). The results of the binding energy analysis supported that coordination bonds between selenium and platinum atom in MON-Pt were formed. The GSH-responsive degradation of MON-Pt inducing controlled drug release was further investigated. The rapid release of cisplatin (>70% after 12 h) was observed in 10 mM GSH solution mimicking the tumor microenvironment (Figure 2I). In PBS solution without GSH, less than 10% of cisplatin is released in 96 h. To further demonstrate the advantages of diselenide-bridged MON for controllable cisplatin delivery, mesoporous silica nanoparticles loading cisplatin (MSN-Pt) were prepared as a comparison (Supplementary Figure S4). The cisplatin loading capacity of MSN (MSN-Pt) was about 4.3% determined by ICP-OES. Compared with MON-Pt, nondegradable MSN-Pt exhibited a slower cisplatin release manner in 10 mM GSH solution (Figure 2I and Supplementary Figure S5), but increased a high degree of premature leakage (40%) after incubation in PBS solution over 48 h (Supplementary Figure S5). These results verify that the coordination bind between selenium and platinum atoms prevented premature drug leakage, facilitating the controlled on-demand release of cisplatin in response to the tumor microenvironment.

### Preparation and Characterization of MON-Pt@CM

**FIGURE 3:**
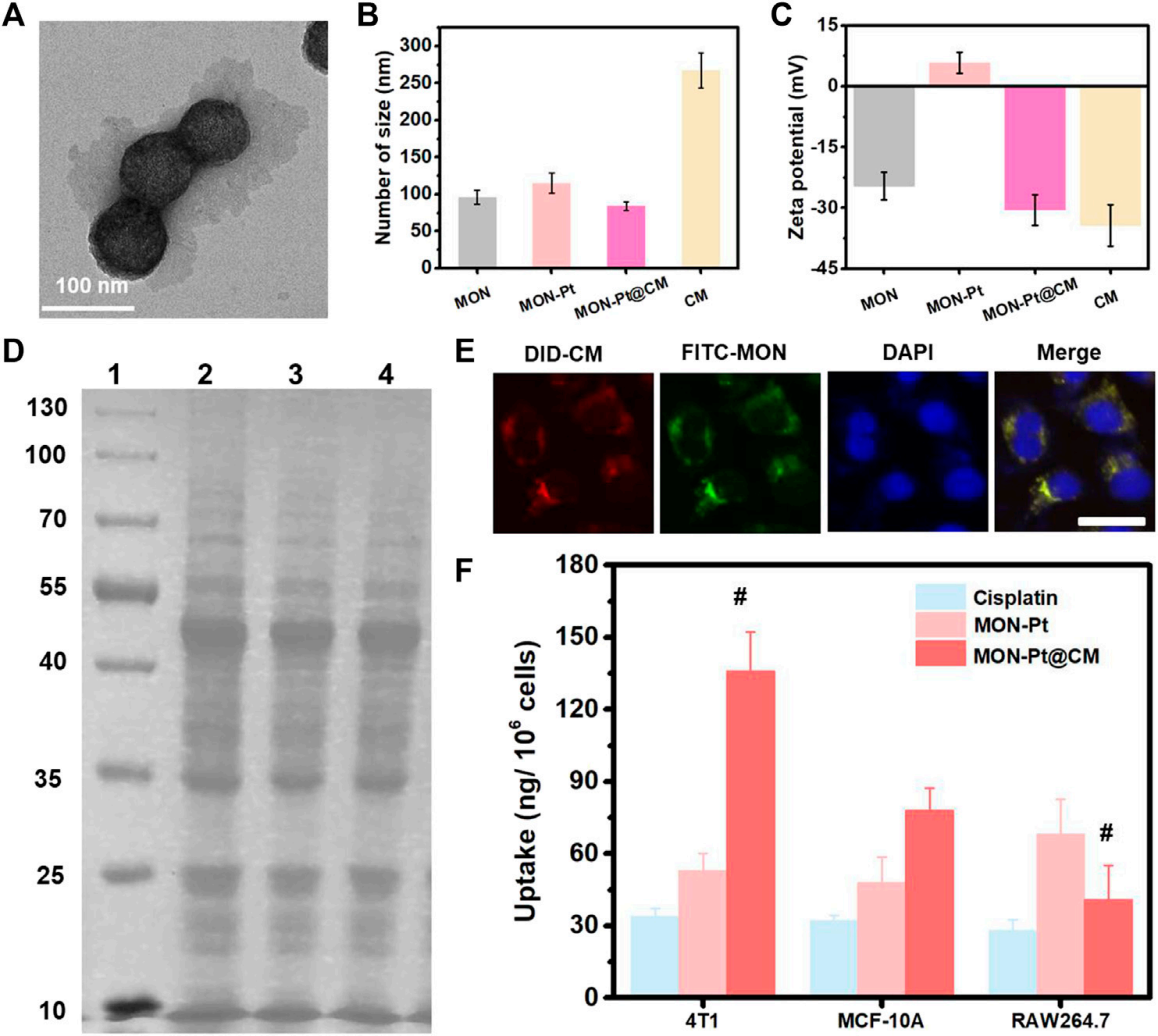
Preparation and characterization of MON-Pt@CM. **(A)** TEM, **(B)** size, **(C)** zeta potential of MON-Pt@CM. **(D)** SDS-PAGE protein analysis. 1-well: marker, 10–130 kDa; 2-well: cell lysate; 3-well: CM vesicle; 4-well: MON-Pt@CM. **(E)** intracellular colocalization of DiD-labeled CM vesicles (red) and FITC-labeled MON (green) of FITC-MON-Pt@DID-CM in 4T1 cells after incubation for 1 h. Scale bars indicate 5 µm. **(F)** Selective uptake of MON-Pt@KP1339 in 4T1, MCF-10A, and RAW264.7 cells after incubation for 6 h ^#^ *p* < .05 compared with MON-Pt group.

Besides on-demand release, targeted drug delivery of cisplatin is another important pathway to overcome unwanted side effects (Shao et al., 2018b; Shao et al., 2020; Kuo et al., 2021). Breast cancer cell membrane (CM) coating nanocarriers could realize homologous tumor-targeted and immune-evasive drugs delivery. 4T1 breast cancer cell membrane (CM) was used to coat MON-Pt according to our previous work (Shao et al., 2018a; Zhang et al., 2021). The biomimetic CM-coated MON-Pt (MON-Pt@CM) exhibited a spherical structure with a thin, smooth membrane shell (Figure 3A). The hydrodynamic diameter of MON-Pt@CM was slightly decreased than MON-Pt (Figure 3B) due to the CM coating facilitating the stability while the surface charge property was decreased dramatically and similar to the CM vesicles (Figure 3C). These results confirm that we successfully constructed a CM-coated MON-Pt. The MON-Pt@CM showed excellent colloidal stability incubating in 10% fetal bovine serum-containing medium over 7 days, and MON-Pt showed significant aggregation (Supplementary Figure S6). These results indicate that CM coating promoted colloidal stability. Protein electrophoresis analysis further suggests that the membrane proteins from 4T1 CM proteins could be well retained on the MON-Pt@CM (Figure 3D). 4T1 breast cells incubated with dye-labeled FITC-MON-Pt@ DiD-CM for 3 h. A high degree of intracellular colocalization indicated the structural integrity of CM-cloaked MON-Pt during the delivery process (Figure 3E), which benefited from keeping homologous tumor-targeted and immune-evasive from CM. Higher internalization of MON-Pt@CM was observed in 4T1 cells than in MCF-10A or RAW264.7 macrophages (Figure 3F). The facts suggest that biomimetic MON-Pt@CM have homologous targeting ability and immune system evasion advantages. MON-Pt@CM anti-tumor and GSH depletion *in vitro*.

**FIGURE 4:**
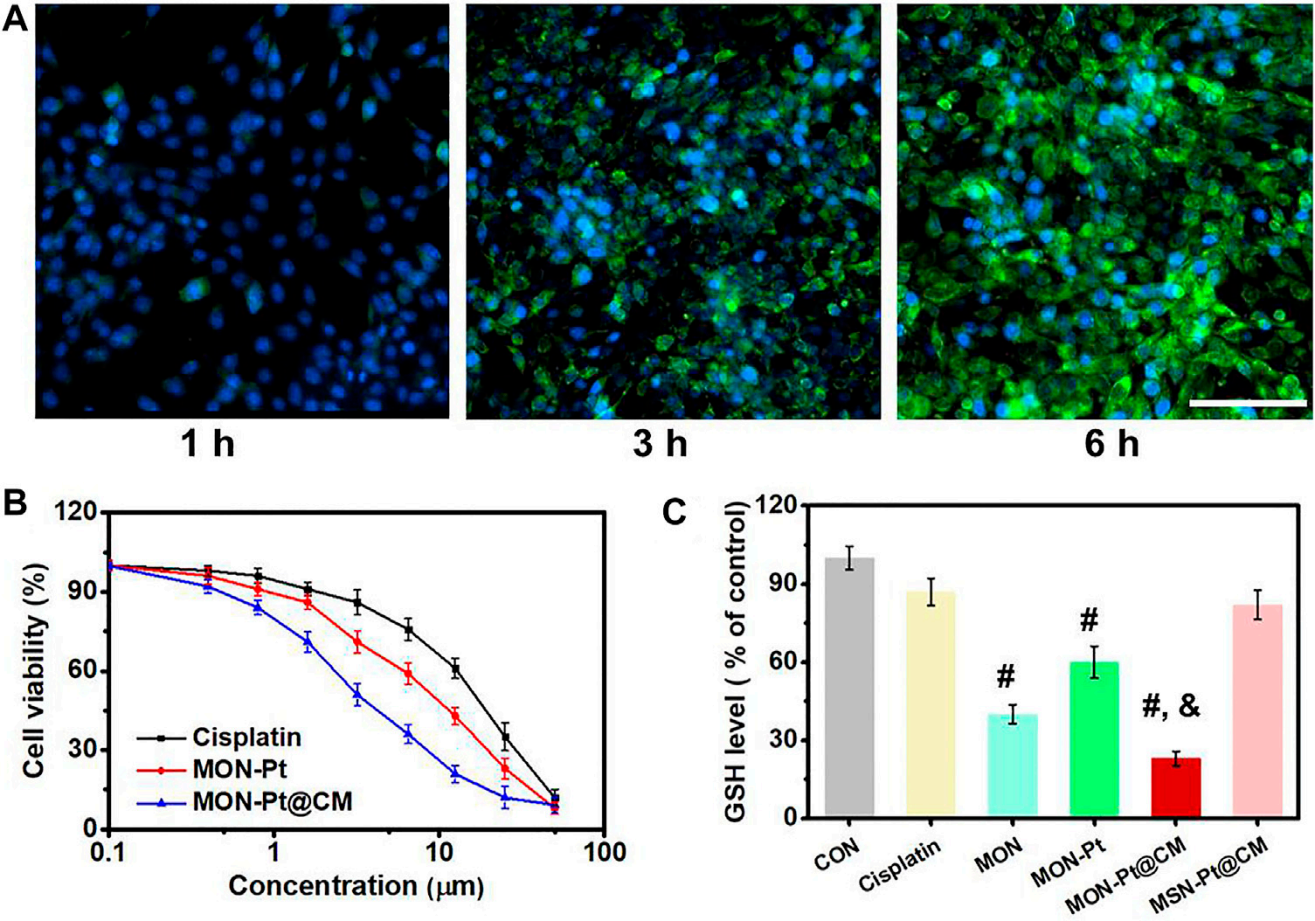
MON-Pt@CM antitumor and GSH depletion *in vitro*. **(A)** Uptake of 4T1 cells incubated with FITC-MON-Pt@CM. Scale bars indicate 50 µm. **(B)** Cytotoxicity of MON-Pt@CM. **(C)** GSH depletion of MON-Pt@CM in 4T1 cells. ^#, &^ *p* < .05 compared with cisplatin group (^#^) and MSN-Pt@CM group (^&^).

After 4T1 cells incubated with FITC-labeled, intracellular fluorescence became stronger along with prolonged incubation time (Figure 4A). It implies that MON-Pt@CM could have efficient uptake by 4T1 cells. Given the selective and efficient cellular uptake capacity, the cytotoxicity of MON-Pt@CM against 4T1 cells was subsequently examined by MTT assay. As shown in Figure 4B, free cisplatin, MON-Pt, and MON-Pt@CM all displayed dose-dependent cytotoxicity. It is worth noting that the IC_50_ (the half-maximal inhibitory concentration of drug) of free cisplatin, MON-Pt, and MON-Pt@CM was 15.95, 10.72, and 4.59 μM, respectively. Cell-membrane coating MON-Pt@CM exhibited a stronger antitumor ability. A different mechanism may cause improved cytotoxicity. As expected, biomimetic structure facilitated uptake of cisplatin (Figure 3F). In addition, the cellular concentration of GSH was determined to be obviously reduced after MON-Pt and MON-Pt@CM treatment (Figure 4C). MON was functional modification of PEG on the surface, which could be more stable than MON-Pt in culture medium. The high dispersity may facilitate the uptake of MON to scavenge intracellular GSH. When 4T1 cells were cultured in 5 mM GSH containing medium, the cytotoxicity of cisplatin against 4T1 cells was obvious deactivation. Diselenide bonds containing the MON-Pt@CM nanosystem could reduce the deactivation cisplatin, and enhance antitumor efficiency (Supplementary Figure S7). The results further prove that biomimetic MON-Pt@CM containing the diselenide bond could enhance cisplatin-based chemotherapy. MON-Pt@CM mediated chemotherapy *in vivo*.

A challenge in cisplatin-based chemotherapy is that cisplatin drugs achieve a long blood circulation time and tumor-targeted delivery. To investigate the role of cancer cell camouflaging on cisplatin delivery, we measured pharmacokinetic profiles and biodistribution in mice. The biomimetic MON-Pt@CM exhibited a prolonged elimination half-life (18.4 h) than that of MON-Pt (6.6 h) and free cisplatin (3.7 h) as shown in Supplementary Figure S8. The extended blood circulation time suggests that CM coating conferred colloidal stability and the immune-evasive ability. We examined the biodistribution by determining the platinum content of tumor tissue and major organs (Supplementary Figure S9). Compared with MON-Pt, MON-Pt@CM dramatically reduced liver and spleen retention, indicating that cancer-cell-membrane camouflaging promoted immune cell evasion *in vivo*. Moreover, the biomimetic cancer-cell-membrane of MON-Pt@CM referred homologous targeting capacity, dramatically enhancing tumor accumulation.

**FIGURE 5:**
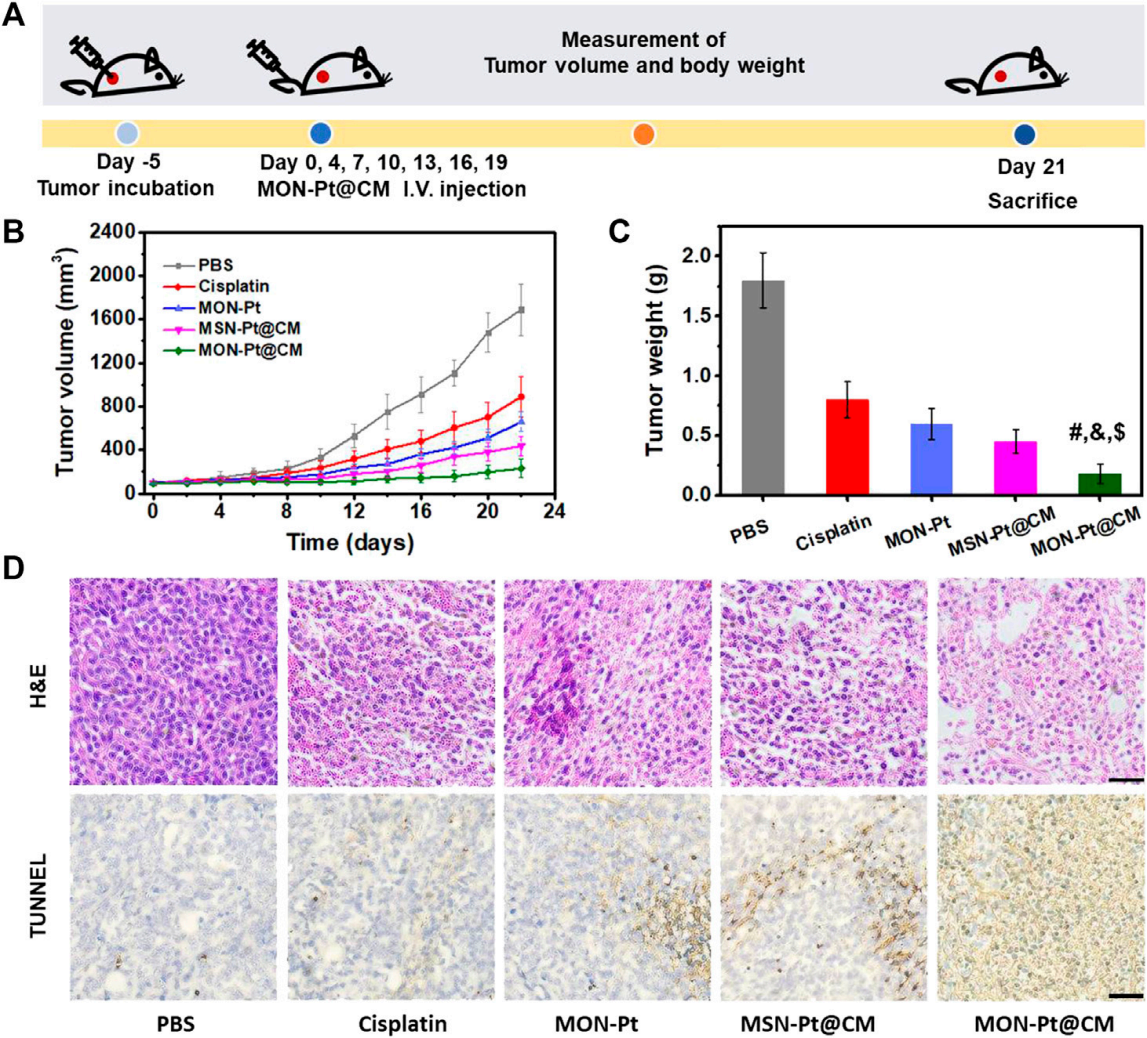
MON-Pt@CM mediated chemotherapy *in vivo*. **(A)** Schematic of treatment schedule in 4T1 bearing tumor model. **(B)** Tumor volume and **(C)** tumor weight. **(D)** H&E stain and TUNEL positive cells of the tumor. Scale bars indicate 50 µm ^#, &, $^ *p* < .05 compared with PBS group (^$^), cisplatin group (^#^), and MSN-Pt@CM group (^&^).

Encouraged by *in vitro* and *in vivo* results, we next evaluated the therapeutic efficacy on mice bearing 4T1 heterotopic mammary tumor (Figure 5A). All mice treated with cisplatin or cisplatin-based formulations showed reduced tumor volumes and tumor weights compared with the control group after treatment (Figures 5B,C, and Supplementary Figure S10). As envisioned, CM coating MON-Pt@CM significantly enhanced the anticancer effect more than MON-Pt, indicating the advantages of homogeneous tumor targeting. To reveal the advantages of diselenide-bridged MON in the delivery of cisplatin, we prepared CM coating MSN-Pt (MSN-Pt@CM), which serves as a control. MON-Pt@CM exhibits a more pleasing anticancer effect than that of MSN-Pt@CM. This phenomenon could be explained by the GSH-responsive release and intracellular GSH deletion to reduce the deactivation of cisplatin. Terminal deoxynucleotidyl transferase dUTP nick end labeling (TUNEL) staining and hematoxylin-eosin (H&E) staining of the tumor sections further revealed the highest apoptosis and necrosis of the tumor cells in MON-Pt@CM groups (Figure 5D).

The safety of cisplatin-based chemotherapy is a significant consideration. None of the mice show apparent weight loss (Supplementary Figure S11). The systemic toxicity was further evaluated by serum biochemical parameters, and no abnormal changes were observed (Supplementary Figure S11). Moreover, H&E staining demonstrated that all treatment groups have no significant pathological changes in the heart, liver, spleen, lung, and kidney (Supplementary Figure S12). Overall, the cancer-cell-membrane-cloaked MON-Pt@CM displayed low systemic toxicity *in vivo*.

In summary, we developed a facile and effective cisplatin drug delivery by diselenide-bond-bridged MONs nanocarrier for safe and efficient chemotherapy. Biodegradable MON was used for activated cisplatin loading by coordination attachment to prevent premature leakage in normal tissue and to achieve degradation-controlled cisplatin release in response to the tumor microenvironment. Besides this, diselenide-bond-bridged MON could consume excess abundant intracellular GSH in the degradation process, which avoids cisplatin deactivation to enhance the chemotherapy effect. MON-Pt coating with cancer CM achieved better tumor targeting and immune system evasion, combined with controlled cisplatin release in response to the tumor microenvironment, to reduce the unwanted side effects. After systemic administration, the MON-Pt@CM exhibited long time in blood circulation and targeting tumor accumulation, leading to remarkable tumor growth inhibition without systematic toxicity. Collectively, this work suggests a biomimetic and biodegradable nanocarrier and offers great promise in enhancing the efficacy and safety of cisplatin-based chemotherapy.

## Data Availability

The original contributions presented in the study are included in the article/Supplementary Material, further inquiries can be directed to the corresponding authors.
