# Supplementary material for: Biomimetic Redox-Responsive Mesoporous Organosilica Nanoparticles Enhance Cisplatin-Based Chemotherapy
Source: Front Bioeng Biotechnol. 2022 Mar 16;10:860949. doi: 10.3389/fbioe.2022.860949 (PMC8966698; doi:10.3389/fbioe.2022.860949)
Supplement: Supplementary file 1 [file DataSheet1.docx]

**Biomimetic Redox-Responsive Mesoporous Organosilica Nanoparticles Enhance Cisplatin-based Chemotherapy**

Fangman Chen^1, 2^, Fan Zhang^2^, Yanbin Wang^3^, Jiahui Peng^1, 2^, Lei Cao^1, 2^, Qian Mei^2^, Mingfeng Ge^2^, Li Li^2^, Meiwan Chen^4^, Wen-fei Dong^1, 2, *^, and Zhimin Chang^2, *^

^1^ School of Biomedical Engineering (Suzhou), Division of Life Sciences and Medicine, University of Science and Technology of China, 96 Jinzhai Road, Hefei 230026, China

^2^ CAS Key Laboratory of Bio Medical Diagnostics, Suzhou Institute of Biomedical Engineering and Technology Chinese Academy of Sciences, 88 Keling Road, Suzhou 215163, China

^3^ Nephrology Department of the Fourth Affiliated Hospital of XinJiang Medical University

^4^ State Key Laboratory of Quality Research in Chinese Medicine, Institute of Chinese Medical Sciences, University of Macau, Macau, China

*Correspondence:

Prof. Wen-fei Dong, School of Biomedical Engineering (Suzhou), Division of Life Sciences and Medicine, University of Science and Technology of China, 96 Jinzhai Road, Hefei 230026, China. E-mail addresses: wenfeidong@sibet.ac.cn

Prof. Zhimin Chang, CAS Key Laboratory of Bio Medical Diagnostics, Suzhou Institute of Biomedical Engineering and Technology Chinese Academy of Sciences, 88 Keling Road, Suzhou 215163, China. E-mail: changzm@sibet.ac.cn.

3.1 Chemicals and Reagents

Sigma-Aldrich (St. Louis, MO, United States) provided tetraethyl orthosilicate (TEOS), silver nitrate (AgNO_3_), aluminum-tri-sec-butoxide, triethylamine (TEA), cisplatin, and. Beijing Chemical Reagent Co. (Beijing China) supplied the Bis[3-(triethoxysilyl)propyl]tetrasulfide (BTESPT), fluorescein isothiocyanate (FITC), and anhydrous ethanol. GIBCO (Carlsbad, CA, United States) offered the [3-(4, 5-dimethylthiazol-2-yl)-2, 5-diphenyl] tetrazolium bromide (MTT), Dulbecco’s Modified Eagle Medium (DMEM), fetal bovine serum (FBS), penicillinstreptomycin, and trypsin (10,000 U/mL). Matrigel was purchased from Corning Inc. (Billerica, MA, United States). The Ellman's Reagent, reduced GSH assay kit, 4, 6-diamidino-2-phenylindole (DAPI), 4-chlorobenzenesulfonate salt (DiD), and LysoTracker Red DND-99 were obtained from Thermo Fisher Scientific (Waltham, MA, United States). Assay kits to determine alkaline phosphatase (ALP), alanine aminotransferase (ALT), aspartate aminotransferase (AST), blood urea nitrogen (BUN) and creatinine (CRE) were purchased from the Nanjing Jiancheng Bioengineering Institute (Nanjing, Jiangsu, China). The above reagents were directly applied with no purification.

3.5 Characterization and degradation

The morphologies of the MON were characterized with a JEM-2100F transmission electron microscope (TEM, JEOL, Ltd., Japan) and a scanning electron microscope (SEM, FEI Quanta 200F). Energy-dispersive X-ray spectroscopy (EDX) was performed with a JEM-2100F EDX system. The hydrodynamic diameter and zeta potential of the NPs were characterized with a Nano-ZS 90 Nanosizer (Malvern Instruments Ltd., Worcestershire, UK). UV-vis adsorption spectra were recorded on a U-3310 spectrophotometer (Hitachi, Japan). Specific surface area and pore size distributions were evaluated and calculated by the Brunauer-Emmett-Teller (BET) and Barrett-Joyner-Halenda (BJH) methods. High-resolution XPS spectra data were collected with a VG ESCALAB MKII spectrometer with an Mg KR excitation (1253.6 eV). Binding energy calibration was based on C 1s at 284.6 eV. Platinum and Se content was measured by ICP-OES.

SDS-PAGE was used to characterize the protein composition of CM@MON@DOX. The marker protein CD47 of 4T1 cells in 4T1 cell membranes and CM@MON@DOX was identified by Western blot. Stability experiments were performed by measuring nanoparticles in DMEM with 10% FBS for 7 days using a Nano-ZS 90 Nanosizer. Degradation of MON (100 μg·mL^-1^) was evaluated in DI water with 10 mM GSH. The solution was incubated at 37 °C under constant rotation. Samples were collected for TEM at 0, 1 and 3 days.

1.1 Statistical Analysis

All experiments were repeated at least three times, and the outcomes were displayed as means ± standard deviations. A contrast between the groups was calculated using a Student’s t-test (two groups) or Bonferroni’s post hoc test (three groups or more). Differences were considered statistically significant when the p-values were less than 0.05.


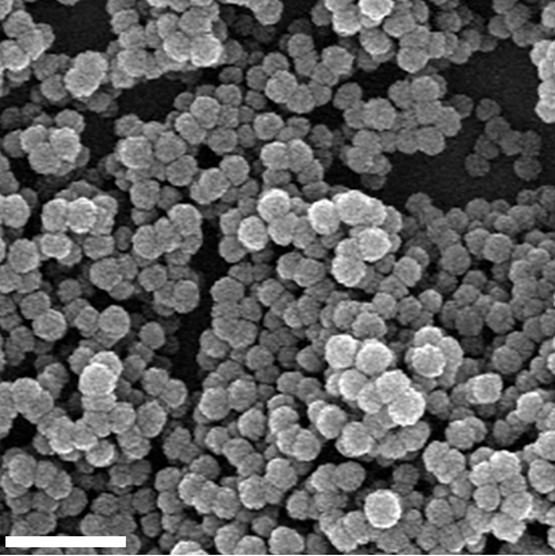


**Figure S1.** SEM image of MON.


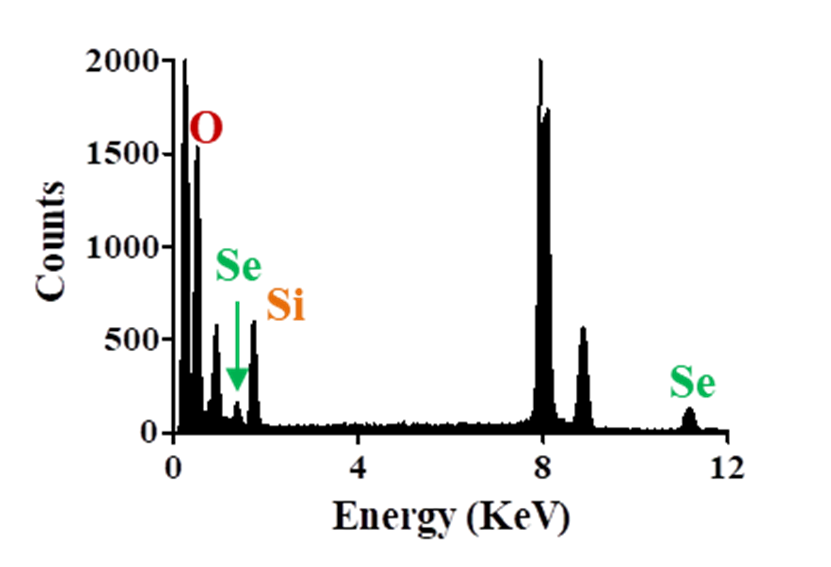


**Figure S2.** EDS spectrogram of MON.


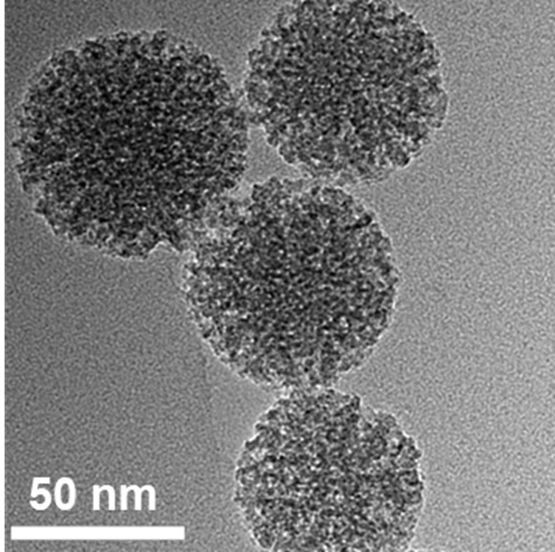


**Figure S3.** TEM image of MON after 3 d incubation with PBS


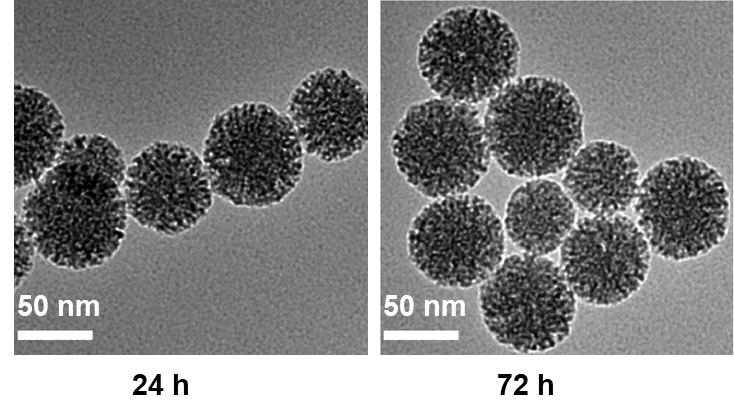


**Figure S4.** TEM image of MSN after 1 and 3 d incubation with GSH solution.


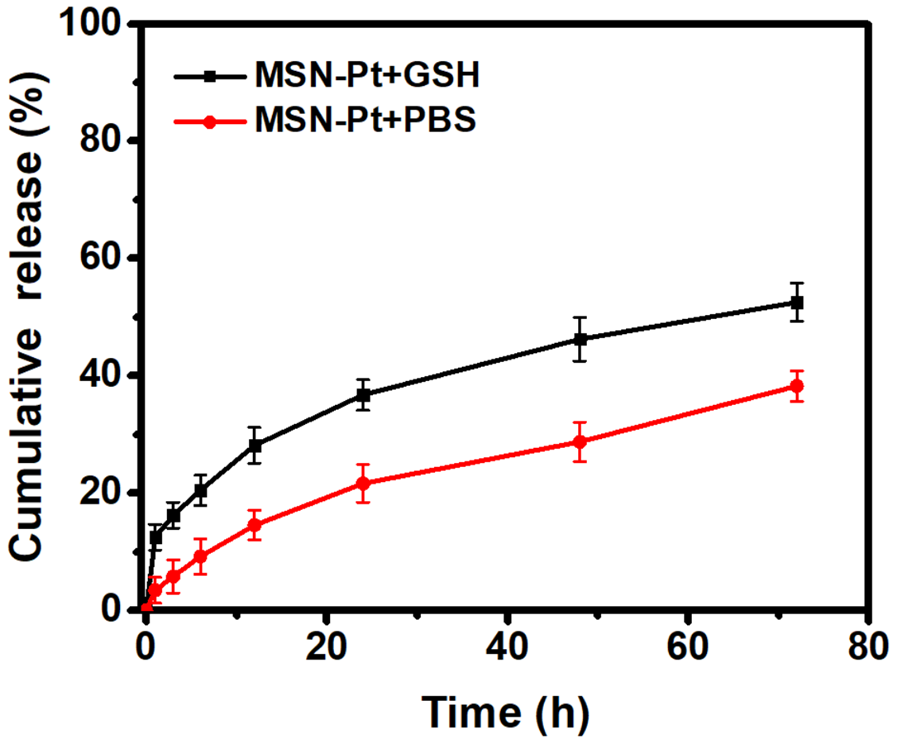


**Figure S5.** Cisplatin release profiles of MSN-Pt in the present or absent of 10 mM GSH. All data are mean ± SD (n=3)


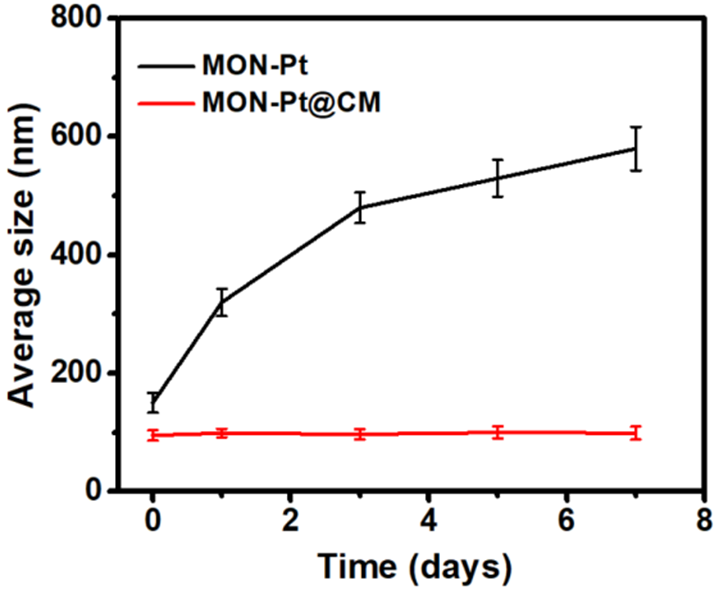


**Figure S6.** Colloidal stability of MON-Pt@CM in DMEM plus 10% FBS for 7 days.


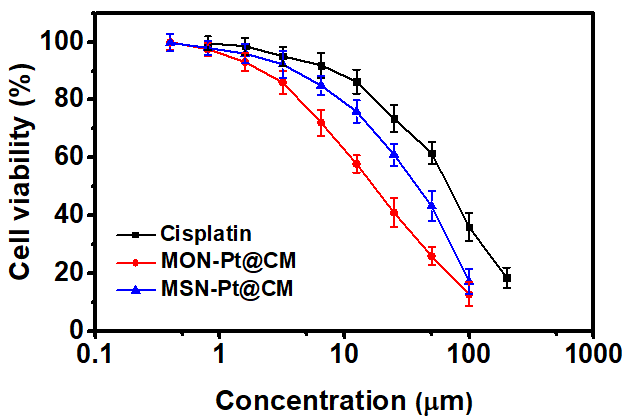


**Figure S7.** Cytotoxicity of cisplatin, MON-Pt@CM and MON-Pt@CM against 4T1 cells in 10mM GSH containing medium.


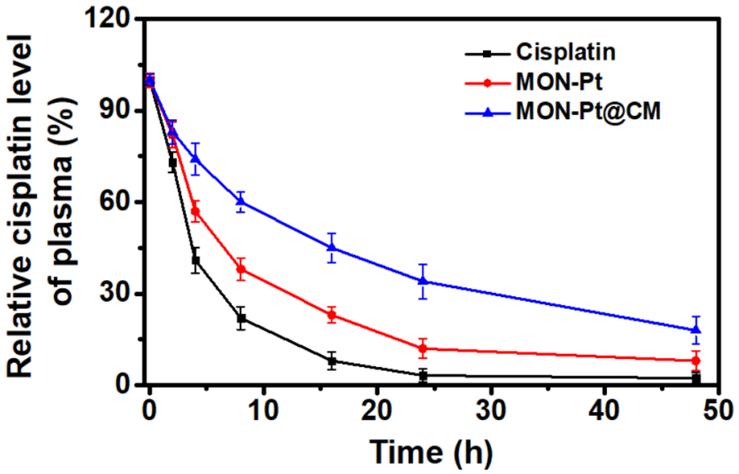


**Figure S8.** Blood circulation time MON-Pt@CM uncoated MON-Pt, and free cisplatin in 4T1-tumor-bearing mice. All data are mean ± SD (n = 5).


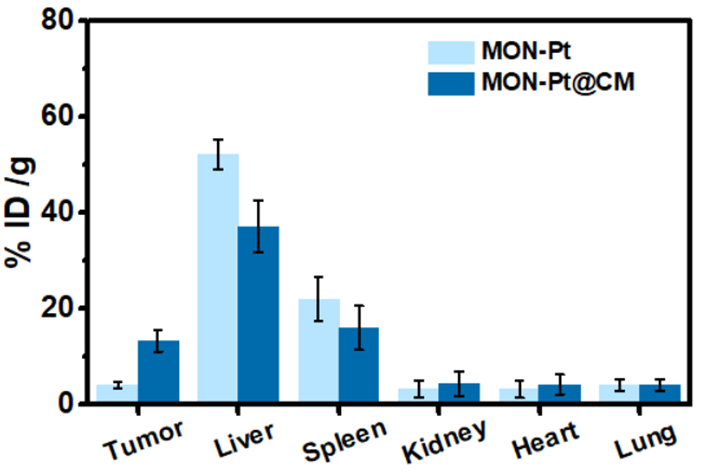


**Figure S9.** biodistribution of MON-Pt@CM and uncoated MON-Pt in 4T1-tumor-bearing mice. All data are mean ± SD (n = 5).


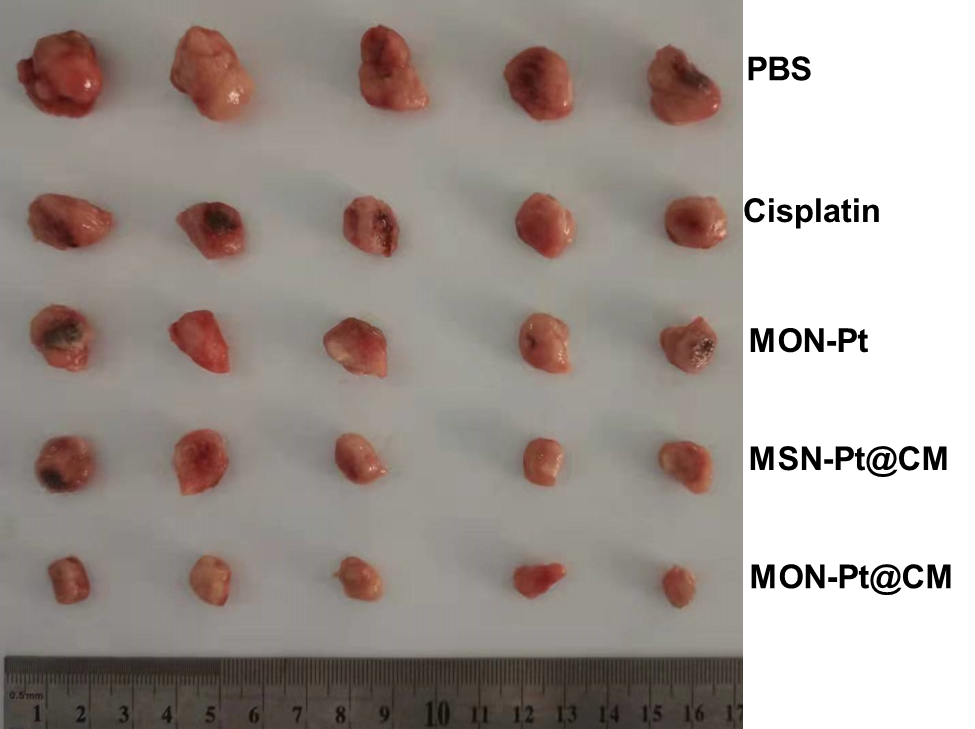


**Figure S10.** Tumor photographs of MON-Pt@CM-treated 4T1 tumor-bearing mice over 21 days. Data represent the mean ± SD (n=5).


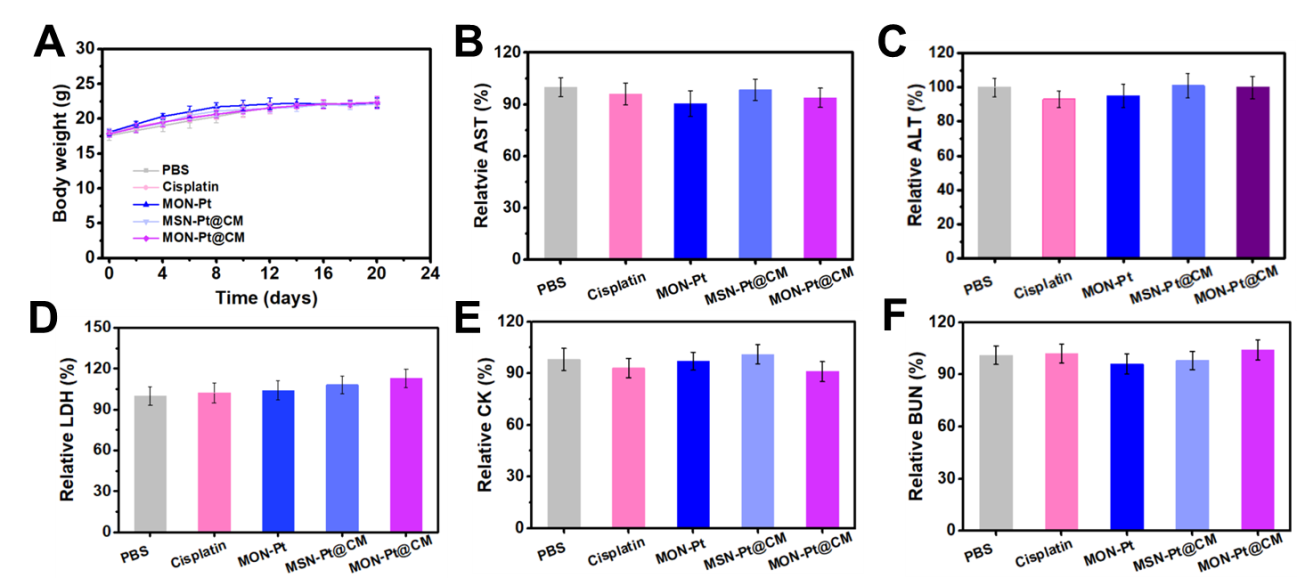


**Figure S11.** (A) Body weight and biochemical parameters including (B) aspartate aminotransferase (AST), (C) alanine aminotransferase (ALT), (D) lactate dehydrogenase (LDH), (E) creatinine kinase (CK), (F) blood urea nitrogen (BUN) of 4T1 tumor-bearing mice after 21-day of treatment. Data represent the mean ± SD (n=5).


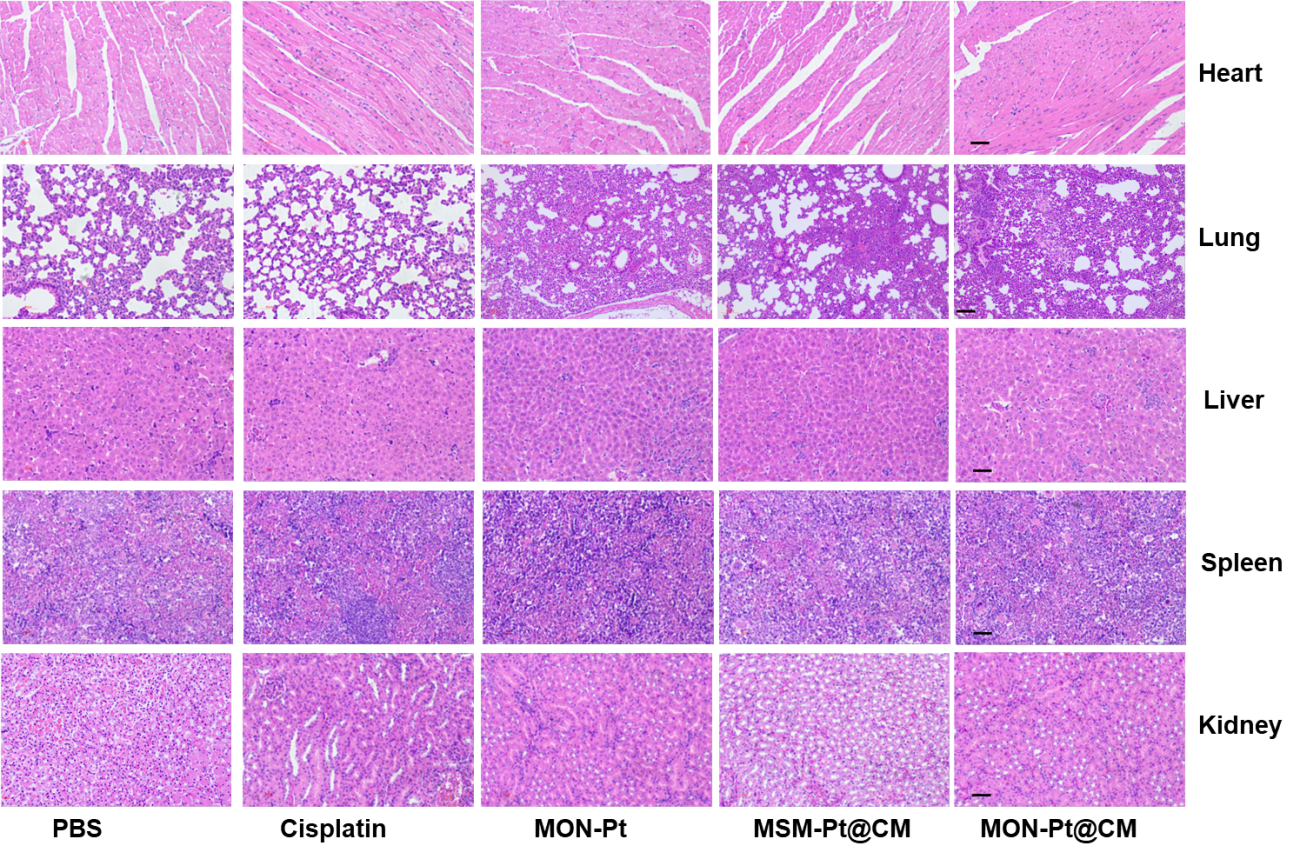


**Figure S12.** Histological evaluation from the major organs including liver, spleen, kidney, heart, and lung of 4T1 tumor-bearing mice after 21-day of treatment. Scale bars represent 50 μm.
